# Supplementary material for: MetaRibo-Seq measures translation in microbiomes
Source: Nat Commun. 2020 Jun 29;11:3268. doi: 10.1038/s41467-020-17081-z (PMC7324362; doi:10.1038/s41467-020-17081-z)
Supplement: Supplementary file 10 — Supplementary Data 7 [file 41467_2020_17081_MOESM10_ESM.zip › File2/Confidence_VeryHigh_Taxonomy/133687_out.krona.html]

Javascript must be enabled to view this page.

members
magnitude
magnitudeUnassigned
count
unassigned
taxon
rank

133687\_out

5

superkingdom
5
2

phylum
1239
5

909932
5
class

5
1843489
order

family
5
31977

genus
39948
5

5
2161821

SRS013521\_contig\_number\_10972SRS018541\_contig\_number\_contig-100\_1194.92927SRS046502\_contig\_number\_11057SRS1041039\_contig\_number\_9813SRS1041137\_contig\_number\_27936
species
